# Multiple Facets of cAMP Signalling and Physiological Impact: cAMP Compartmentalization in the Lung

**DOI:** 10.3390/ph5121291

**Published:** 2012-11-29

**Authors:** Anouk Oldenburger, Harm Maarsingh, Martina Schmidt

**Affiliations:** 1 Department of Molecular Pharmacology, Groningen Research Institute for Pharmacy, University of Groningen, 9713 AV, Groningen, The Netherlands; 2 Groningen Research Institute for Asthma and COPD, University of Groningen, University Medical Center Groningen, 9700 RB, Groningen, The Netherlands

**Keywords:** cAMP compartmentalization, barrier function, COPD, A-kinase anchoring proteins (AKAPs), Epac

## Abstract

Therapies involving elevation of the endogenous suppressor cyclic AMP (cAMP) are currently used in the treatment of several chronic inflammatory disorders, including chronic obstructive pulmonary disease (COPD). Characteristics of COPD are airway obstruction, airway inflammation and airway remodelling, processes encompassed by increased airway smooth muscle mass, epithelial changes, goblet cell and submucosal gland hyperplasia. In addition to inflammatory cells, airway smooth muscle cells and (myo)fibroblasts, epithelial cells underpin a variety of key responses in the airways such as inflammatory cytokine release, airway remodelling, mucus hypersecretion and airway barrier function. Cigarette smoke, being next to environmental pollution the main cause of COPD, is believed to cause epithelial hyperpermeability by disrupting the barrier function. Here we will focus on the most recent progress on compartmentalized signalling by cAMP. In addition to G protein-coupled receptors, adenylyl cyclases, cAMP-specific phospho-diesterases (PDEs) maintain compartmentalized cAMP signalling. Intriguingly, spatially discrete cAMP-sensing signalling complexes seem also to involve distinct members of the A-kinase anchoring (AKAP) superfamily and IQ motif containing GTPase activating protein (IQGAPs). In this review, we will highlight the interaction between cAMP and the epithelial barrier to retain proper lung function and to alleviate COPD symptoms and focus on the possible molecular mechanisms involved in this process. Future studies should include the development of cAMP-sensing multiprotein complex specific disruptors and/or stabilizers to orchestrate cellular functions. Compartmentalized cAMP signalling regulates important cellular processes in the lung and may serve as a therapeutic target.

## 1. Introduction

**Figure 1:**
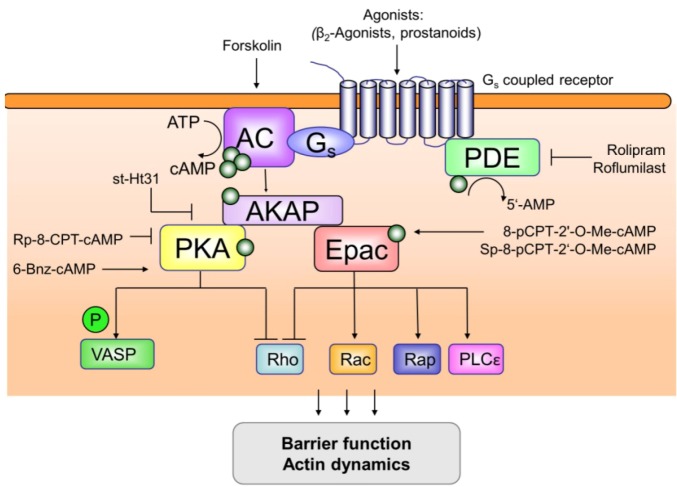
Overview of compartmentalization of cAMP signalling. G_s_-protein coupled receptors are stimulated by their appropriate ligands such as β_2_-agonists and prostanoids. Subsequently, activation of adenylyl cyclase (AC) will lead to the production of the second messenger cyclic AMP (cAMP), whereas cAMP-specific phosphodiesterases (PDEs) will shape the cAMP gradient throughout the cell. Alternatively, AC can be directly activated by the cell membrane-permeable diterpene forskolin from the Indian plant *Coleus forskolhlii*. Elevation of cellular cAMP will simultaneously induce the activation of protein kinase A (PKA) and of the exchange protein directly activated by cAMP (Epac). Members of the A-kinase anchoring protein (AKAP) family will support the maintenance of cAMP compartmentalization upon binding to the cAMP-producing receptors, the cAMP effectors PKA and/or Epac as well as PDEs. The generation of cAMP-sensing multiprotein complexes by AKAPs is of tremendous importance to maintain spatio-temporal cAMP signalling at specific and discrete locations within the cell to regulate specific cellular responses upon signalling to several distinct effector proteins including vasodilator-stimulated phosphoprotein (VASP), a subset of small GTPases, and phospholipase C-ε (PLC-ε). Shown are tools being used to study the functioning of the cAMP-sensing multiprotein complexes: st-Ht31, the PKA binding blocking peptide known to act as a generic AKAP inhibitor [14,15,16]; 8-pCPT-2'-O-Me-cAMP and/or Sp-8-pCPT-2'-O-Me-cAMP, activator of Epac; 6-Bnz-cAMP, activator of PKA; Rp-8-CPT-cAMP, Rp-cAMPs, Rp-8-Bromo-cAMPs inhibitors of PKA.

Cyclic adenosine monophosphate (cAMP), the most common and universal secondary messenger, regulates physiological processes as diverse as calcium handling, secretion, ion channel conductance, learning and memory, metabolic events, cardiac and smooth muscle contraction, cell growth and differentiation, apoptosis, inflammation, and barrier functioning [1,2]. The impact and complexity of research into the molecular architecture of cAMP signalling is not only reflected by five Nobel awards since the discovery of cAMP in 1957 by Sutherland and colleagues [1], but also by a unique interplay of signalling components that tightly control the cellular content of cAMP. Next to G protein-coupled receptors, adenylyl cyclases (ACs) and cAMP-specific phosphodiesterases (PDEs) maintain the spatio-temporal nature of cAMP signalling by shaping a cAMP gradient throughout the cell [3,4,5]. Subcellular membrane clustering of receptors, ACs and PDEs to lipid rafts and caveolae [6,7,8], and cell compartment-specific (co)localization to distinct cAMP effectors [9,10,11,12,13] further support the maintenance of spatio-temporal compartmentalized cAMP signalling. Moreover, A-kinase anchoring proteins (AKAPs) facilitate subcellular cAMP spatio-temporal compartmentalization by generating spatially discrete signalling complexes that create local gradients of cAMP, and thereby permit and control specific cellular responses (Figure 1) [14,15,16,17]. Dysfunctions of cAMP-sensing AKAP complexes seem to contribute to the progression of a wide variety of diseases, including chronic heart failure, cardiac arrhythmia, Alzheimer’s dementia, HIV infection, diabetes mellitus and cancer [17,18,19,20,21], hence, current research intends to target the spatio-temporal cAMP-responsive complexes to provide novel therapeutical interventions [5,11,12,17,22]. 

In this review we will discuss the recent progress on spatio-temporal compartmentalized cAMP signalling from the receptors coupled to the cAMP pathway up to the subtle interplay between the distinct cAMP-sensitive effectors that maintain cAMP-sensing multiprotein complexes. In particular, we will focus on the impact of perturbation of cAMP-sensing signalling complexes in the development and progression of chronic obstructive pulmonary disease (COPD), a chronic inflammatory lung disease characterized by airway obstruction, emphysema and airway remodelling. Remodelling processes encompass increased airway smooth muscle mass, epithelial changes, goblet cell and submucosal gland hyperplasia—leading to mucus hypersecretion [23,24,25,26,27,28,29,30]. In addition to inflammatory cells, airway smooth muscle cells and (myo)fibroblasts, epithelial cells underpin a variety of key responses in the airways such as inflammatory cytokine release, airway remodelling, mucus hypersecretion and the barrier function [23,24,25,26,27,28,29,30]. Cigarette smoke—together with environmental pollution—is the main risk factor for COPD and induces inflammatory processes, alveolar destruction (emphysema), fibrosis and epithelial hyperpermeability by disrupting the barrier function, releasing proteases and inducing multiple inflammatory genes [27,28,29,31]. Disruption of the epithelial barrier is associated with epithelial remodelling that also accounts for goblet cell metaplasia and mucus gland hypertrophy in COPD [32,33]. Mucus hypersecretion contributes to the morbidity and mortality of COPD, particularly in those patients with more severe disease [27,28,34]. In the treatment of obstructive lung diseases, including COPD, cAMP elevating drugs are widely used. Already in the early eighties it has been reported that cAMP elevating agents, such as β_2_-agonists, prostanoids and the direct AC activator forskolin (Figure 1), temper oedema in whole animal, isolated lung, and clinical studies of lung injury, phenomena which could be linked to an increase in barrier function in pulmonary endothelial cells [2].

Our current knowledge, however, about the molecular mechanisms underlying proper epithelial barrier functioning in the airways is mainly based on studies with focus on the endothelial barrier in the vasculature [2]. For the purpose of this review, we will outline our current knowledge about compartmentalized cAMP signalling. We will highlight the role of the epithelial barrier to maintain proper lung functioning and to alleviate COPD symptoms. The regulation of the endothelial barrier will serve as a starting point, and whenever appropriate, we will focus on the epithelial barrier function.

## 2. Spatio-Temporal Nature of Compartmentalized cAMP Signalling: Paradigm Shifts

The formation of cAMP is initiated by the stimulation of G_s_-protein-coupled receptors, such as the β_2_-adrenoceptor and distinct prostanoid receptor subtypes. As members of the largest superfamily of cell surface signalling molecules, cAMP-elevating G_s_-protein-coupled receptors represent the most prominent family of validated pharmacological targets in biomedicine [1,35,36]. In obstructive airways diseases short- and long-acting β_2_-agonists, such as salbutamol/albuterol, fenoterol, formoterol and indaceterol, are clinically widely used and act via stimulation of G_s_-protein-coupled receptors [37,38,39,40]. In addition, recent studies emphasize also substantial progress to pharmacologically target the prostanoid PGE_2_-receptors to alleviate symptoms of obstructive lung diseases [41,42,43,44,45]. Over the last years substantial progress has been made to decipher the distinct signalling properties of cAMP. Initially, elevation of cellular cAMP by β_2_-agonists and prostanoids were expected to simultaneously stimulate both protein kinase A (PKA) and the exchange protein directly activated by cAMP (Epac) [1,46,47]. Meanwhile, it is generally accepted that spatio-temporal compartmentalization of cAMP maintained by cAMP-sensing AKAP-bearing multiprotein complexes and PDEs is of utmost importance to gain signalling specificity of cAMP [4,9,10,11,12,13,14,15,16,17].

Generally, G protein coupled receptors are considered as cell surface recognition sites sensing ions, hormones, neurotransmitters, autocoids and extracellular matrix components [36,38,48,49,50]. More recent studies showed that also internalized G protein-coupled receptors—until now believed to act as a ‘loss-of-function’ receptor signal—maintain signalling properties [18,51,52,53]. Using fluorescence resonance energy transfer to track intracellular cAMP fluctuations following activation of typically G_s_-protein-coupled receptors [51,52], it has been reported that AC signalling is not necessarily restricted to the plasma membrane, but could be also detected in the endosome compartment. Indeed, endosomes, in which internalized receptors may end up, are now recognized as essential sites of cellular signalling [54,55], In addition, actin-stabilized endosomal microdomains profoundly affect the endosomal recycling and thereby the signalling properties of the β_2_-adrenoceptor [56]. Strikingly, Nikolaev and colleagues demonstrated that the β_2_-adrenoceptor its redistributed in heart failure, thereby compartmentalizing cAMP, a process proposed to contribute to the failing myocardial phenotype [18]. While the novel concept of cAMP signalling by internalized G protein-coupled receptors has recently been adapted to the signalling properties of the β_2_-adrenoceptor in human small airways [37], evidence that such mechanisms are operational in distinct structural airway cell subtypes, including bronchial epithelial cells, still has to be provided. 

Intriguingly, ligand-directed signalling or biased agonism, referring to G_s_-induced cAMP- *versus* β-arrestin-mediated signalling in response to different agonists [48,49,57,58,59], adds another level of complexity of G_s_-protein-coupled receptor signalling, and has recently been reviewed within the context of obstructive lung diseases and the β_2_-adrenoceptor [38,40,60,61,62]. In mice, genetic ablation of either β-arrestin-1 or -2 prevented against bleomycin-induced pulmonary fibrosis and fibroblast invasion, suggesting a role for β-arrestin in fibrosis [63]. In support, β-arrestin-2 expression in increased in cell models of cystic fibrosis as well as in nasal tissue from patients [64]. In human bronchial epithelial cells, β-arrestin is necessary for the transcription of matrix metalloproteinases (MMPs) by diesel exhaust particles, a risk factor for COPD [65]. In addition to modulation of remodelling processes, β-arrestin is involved in agonist-induced desensitisation of the β_2_-receptor by inducing the internalization of this receptor [48]. Lefkowitz and colleagues reported that β-arrestin-mediated signalling exerts an even higher degree of regulation that relies on distinct phosphorylation sites of seven transmembrane receptors [66,67,68]. Likewise, β-arrestin-dependent signalling and trafficking of the β_2_-adrenoceptor also involve an unique deubiquitinase-ligase interplay [69,70]. Although recent studies indicate that ligand-directed signalling contributes to the functional responses of airway smooth muscle cells and lung fibroblasts [71,72], comparable studies in airway epithelial cells are still lacking. Further regulation of G_s_ signalling is mediated by the AKAP family members AKAP5 (aka AKAP79/150) and AKAP12 (aka AKAP250/Gravin), which regulate the de- and resensitization of the β_2_-adrenoceptor, respectively, and interact next to cAMP signalling proteins also with β-arrestin [14,15,16,17,73,74,75]. Thus, it is tempting to speculate that biased agonism might also profoundly alter the functional responses of AKAP-bearing multiprotein complexes. Moreover, receptors that ‘typically’ signal via G_s_, including the β_2_-adrenoceptors, have also been shown to couple to other G-proteins, including G_i_ and G_12/13_, adding another layer of complexity to the regulatory pathways [76].

Recent studies indicate that, next to PDEs and ACs [77], members of the AKAP superfamily are of tremendous importance to maintain compartmentalized cAMP signalling and to prevent the progression of several diseases, such as chronic heart failure, Alzheimer’s dementia and cancer [17,18,19,77]. AKAPs exhibit a distinct (sub)cellular expression pattern and linkage to a diverse subset of target proteins including G_s_-protein coupled receptors and ACs, cAMP effector proteins like PKA and Epac as well as cAMP-degrading PDEs (Figure 1). Cooper and colleagues reported recently that AKAP5 is target of palmitylation-dependent localization to lipid rafts and that the lipid modification of AKAP5 promotes its regulation of the calcium-sensitive AC subtype 8, adding an additional regulatory and targeting option for AKAP members [78]. Based on their binding specificity, cellular expression profiles and cellular localization, AKAPs integrate differential coupling of cAMP to specific cellular responses, including smooth muscle tone, cell proliferation and differentiation, learning and memory, inflammation, fibrosis, and barrier functioning (Figure 1) [14,15,16,17]. Intriguingly, the first cAMP-responsive multiprotein complexes identified in the heart and neurons possess a rather distinct composition: i) the cardiac-specific cAMP-responsive complex is maintained by the *nuclear* envelope-associated mAKAP, PKA, PDE4D3 and Epac1 [79], whereas ii) the neuronal cAMP-sensing complex is maintained by the *plasma membrane*-associated AKAP5, PKA, Epac2 and phosphoinositide 3-kinase-dependent protein kinase B (PKB/Akt) [80]. Generation of distinct cAMP-sensitive multiprotein complexes maintained by AKAP family members might turn out to be the key to explain that even though Epac and PKA can act independently, most cAMP-dependent processes are interconnectively regulated by Epac and PKA. Classically, most cAMP effects were assigned to PKA [81,82,83].The identification of Epac as an cAMP-regulated guanine nucleotide exchange factor (GEF) that favours GDP/GTP exchange and thereby activation of small Ras-like GTPases, profoundly changed the classical cAMP-PKA dogma [84,85]. The cAMP mediators Epac1 (aka cAMP-GEF-I) and Epac2 (aka cAMP-GEF-II) function as molecular links between members of the Ras superfamily such as Rho, Rac and Ras [86,87,88,89,90]. Members of the Ras superfamily belong to the GTP-ases which switch between a GDP-bound (inactive) state and a GTP-bound (active) state. Guanine exchange factors (GEFs) such as Epac, will exchange GDP for GTP, activating the effector. GTPase-activating proteins (GAPs) will reduce GTP-ase activity due to GTP hydrolyzation. GTP-binding of Rho, Rac or Ras will induce a diversity of cellular processes (see Figure 1).

Upon activation of distinct subset of small GTPases, Epac1 and Epac2 signal to phospholipase C-ε [91,92], phospholipase D [90,93], extracellular signal-regulated kinases (ERK1/2) [94,95,96,97,98,99], PKB/Akt [80,99,100,101,102,103,104] and NF-κB [105,106,107], and thereby control distinct cellular responses, including calcium handling, smooth muscle tone, cell proliferation and differentiation, migration, fibrogenic and inflammatory responses as well as barrier functioning [2,46,107,108,109,110]. We would like to refer the reader to excellent recent reviews with focus on the molecular signalling properties of Epac [2,46,47,108,109,110,111,112].

Recent studies reported on the contribution of cAMP-sensing AKAP-bearing multiprotein complexes to functional responses of different cell types of the airways. Human airway smooth muscle express seven of the nine membrane-bound AC subtypes [113]. The AC subtypes 5/6 differentially respond to β_2_-adrenoceptor and prostanoid receptor agonists, and thereby represent key molecules to generate cAMP [113,114,115], a process predominantly tuned by PDE4D5 [3,4,61]. As the expression of PDE4D5 is up-regulated by cAMP on the level of gene expression, protein expression and activity [116], cAMP seems to provide a feed-backward signal to diminish its own signalling properties. As PDE4D5 forms a complex with AKAP5 [74], up-regulation of PDE4D5 may alter the delicate spatio-temporal cAMP compartmentalization in human airway smooth muscle and thereby contribute to the progression of airway obstruction. Penn and colleagues reported very recently on the expression of a distinct subset of AKAPs, particularly AKAP12 and ezrin and its impact on compartmentalized cAMP signalling in human airway smooth muscle [117]. 

Human lung fibroblasts have been reported to express six of the nine membrane-bound AC subtypes [118] (Table 1). As in human airway smooth muscle, the AC subtypes 5/6 generate cAMP [118], whereas PDE4 subtypes hydrolyse cAMP in human lung fibroblasts [119,120,121]. A recent study by Peters-Golden and colleagues showed that the AKAP family member AKAP9 (aka AKAP450) represents a key protein for cAMP compartmentalization in fibrotic lung fibroblasts [122]. They reported that collagen deposition is controlled by a prostaglandin E_2_ (PGE_2_)-sensing AKAP9-PKA-protein phosphatase 2A multiprotein complex [122]. Activation of fibroblasts and subsequent collagen synthesis is inhibited by extracellular anti-fibrotic plasmin by restoring the PGE_2_-sensitivity of the fibroblast-specific AKAP9 (splice variant AKAP450) complex [122]. Intriguingly, AKAP9 (splice variant AKAP450) was found to generate a complex with PDE4 [123]. As selective inhibitors of PDE4 including rolipram, cilomilast and roflumilast are studied in clinical trials or licensed for use in COPD [34,124,125,126], targeting AKAP9-PDE4 complexes might be of benefit for COPD patients. The smallest splice variant of AKAP9, Yotiao can associate with ACs subtypes 1, 2, 3 and 9, leading to more phosphorylation of effectors by PKA [127].

**Table 1:** Expression of elements of cAMP signalling in cells and tissues involved in the pathogenesis of lung diseases.

	Epac	PKA	AKAP	PDE	AC	small GTPases
Bronchial epithelium	Epac1 [128] Epac 1 & 2 [129]	PKA [29]	AKAP9 [130]	++ PDE4, PDE1 [131,132] +- PDE3, PDE5 [131,132] PDE4D [133] PDE3A [134] PDE7A1&2 [135]	AC9 [136,137] AC1, 4, 7, 8 [138] sAC [139]	Rap [140,141,142] Rac [29,141,142,143,144,145] Rap1 [129,143] Rap2
Vascular endothelium	Epac1 [146,147,148,149]	PKA [147]	AKAP9 [149] Gravin [150]	PDE4D [22,147,151] PDE4 [135] PDE3 [135]	Membrane bound [2] Soluble AC [2] AC2, 3, 5, 6 [138]	Rap [143,146,147,149,152] Rac [146,147,152,153,154,155,156,157] Rac1 [158] RRas [147,159]
Airway smooth muscle cells	Epac1 [95,107,129] Epac2 [95,107,129]	PKA [107]	AKAP5, 9, 12 [160] Gravin, ezrin [117,150]	PDE1C, 3, 5A, 7 [135,161] PDE7A1&2 [135]	7 membrane bound subtypes [118] 1, 3-7, 9 [115] 2, 6, 7, 9 [113]	RhoA [87] Rac1 [87] Rap1 [95] Rap2 [95]
Vascular smooth muscle cells	Epac1 [162] Epac2 [162]	PKA [162]	AKAP12 [163]	PDE1(C), 3(A), 5 [135] PDE7A1&2 [135] 1A, 1C, 2A, 3A, 3B, 4A, 4B, 4C, 4D, 5A, 7A, 7B, 8A, 9A, 9B, 10A and 11A [164]	AC1, 2, 3, 4, 6, 7, 9 [165] 2, 3, 5, 6, 7, 8 [138]	Rap1 [166] Rac1 [167] RhoA [168, 169]
	
			
Pulmonary fibroblasts	Epac [72] Epac1 [170] Epac2* [170] *only mRNA not protein	PKA [122]	AKAP9 [122]	PDE4A, B, D [119] 3A&B, 4A5, 4B2, 4C1, 4D3, 7 [171]	6 membrane bound subtypes [118]	Rho A [172] Rac1 [172] Rac2 [172] Rap1 [173]
Inflammatory cells	Epac1 [109]	PKA [109]	Ezrin [174] AKAP9 [175]	PDE4B2 [176] PDE1B, 3A, 7A1, 2, 3 [135] 7A1&7A2 [177]	AC [178] 1, 2, 6, 9 [179] 4, 5, 6, 7, 9, sAC [180] sAC [181]	Rap [182] Ras [182] Rac1 [183] Rho [183]

Since airway smooth muscle cells and lung fibroblast both differentially contribute to chronic inflammation, airway obstruction and airway remodelling in COPD there might be a distinct underlying molecular mechanism for these symptoms in airway smooth muscle cells and lung fibroblast. The diverse complex profile of PDE4 subtypes to AKAP5 in airway smooth muscle compared to AKAP9 in lung fibroblasts may be responsible for this.

In contrast to the substantial progress of our current knowledge on the spatio-temporal compartmentalization of cAMP signalling in human airway smooth muscle and lung fibroblasts including subcellular clustering of receptors, ACs and PDEs, neither expression profiling of AC and PDE subtypes nor identification of cAMP-sensing AKAP-bearing multiprotein complexes in human bronchial and/or alveolar epithelial cells have yet been studied in detail (for an overview see Table 1). Whole lung tissue has been reported to express eight of the nine membrane bound AC subtypes [6,184,185]. In particular, the AC subtype 9 seems to represent the key molecule to generate cAMP and thereby alleviate symptoms of obstructive pulmonary disorders [136,137]. Interestingly, a recent report indicated that production of cAMP by the soluble AC subtype contributes to ciliary beat frequency in fully differentiated ciliated airway epithelial cells [186]. Different PDE isoforms are expressed in different epithelial cells. Whereas whole lung tissue primarily expresses PDE4 [187], primary alveolar A549 cells highly express PDE4 compared to PDE1, PDE3 and PDE5 and primary human bronchial epithelial (HBE) cells equally express PDE4 and PDE1, but express lower levels of PDE3 and PDE5 [131,132]. In contrast to studies in endothelial cells, cAMP-sensing multiprotein complexes maintained by AKAPs have not been studied yet in airway epithelial cells, even though PDE4 is complexed with AKAP9 [123]. In human umbilical vein endothelial cells (HUVECs), Epac1-dependent Rap1 activation and subsequent elevations in cortical actin and VE-cadherin increased barrier function. Parallel to this pathway Epac1-AKAP9 complex is required for microtubule growth, integrin adhesion at cell-cell borders and the endothelial barrier function [149]. In line with the previous finding that AKAP9 controls spatio-temporal cAMP dynamics of PDE4 [123], Maurice and colleagues demonstrated that PDE4D-dependent binding of Epac1 to a VE-cadherin-based signalling complex controls vascular permeability [147]. Cell-cell adhesion and integrin-extracellular matrix interactions also seem to rely on Epac, particularly Epac1 [22,151]. Interestingly, in polarized human Calu-3 airway epithelial cells it has been reported that a functional cAMP diffusion barrier maintained by PDE4D determines the activity of the cystic fibrosis transmembrane conductance regulator (CFTR) chloride channel, indicating that functional responses to cAMP in airway epithelial cells rely on cAMP microdomains even though the existence of cAMP-sensing AKAP-bearing multiprotein complexes have not been studied yet [133]. However, Zaccolo and colleagues demonstrated recently that CFTR regulation in human airway epithelial cells required next to the subcortical cytoskeleton compartmentalized cAMP signalling. High cAMP levels localized at the plasma membrane are needed for PKA-dependent activation of CFTR. Compartmentalization is accomplished by a multi-protein complex with Na/H exchange regulatory factor-1 and ezrin. Next to PKA, Epac has also been shown to activate CFTR in response via activation of Rap2 [188] In models of cystic fibrosis, a disease associated with mutations in the CFTR gene, overexpression of wildtype CFTR resulted in an organised cAMP compartmentalization and restored fluid homeostasis [189]. In bronchial epithelial cells, compartmentalized cAMP signaling at the CFTR is regulated by PDE3A in a macrocomplex. PDE3A decreases cAMP at the membrane, thereby inactivating CFTR [134]. Compartmentalization of cAMP at the CFTR is further maintained by binding of the cAMP efflux transporter multidrug resistance protein-4 to CFTR, which reduces cAMP levels near the plasma membrane and reduces CFTR function [190]. 

In addition, multiprotein complexes supporting compartmentalized cAMP signalling are involved in secretory and proliferative functions in airway smooth muscle cells and are maintained by AKAP5 (aka AKAP79/150) and AKAP12 (aka AKAP250). Cigarette smoke-induced perturbation of airway smooth muscle compartmentalization of cAMP might contribute to the development and progression of COPD [160,191]. To summarize, distinct composition of cAMP-responsive AKAP-bearing multiprotein complexes seem to generate and to maintain local cAMP gradients, and thereby to regulate cell-type specific functions in structural airway cells. 

The functional role of cAMP-signalling complexes can be studied using pharmacological tools. Perturbation of AKAP-bearing complexes can be achieved with the PKA-binding blocking peptides such as st-Ht31 [14,16,17]. Novel developed AKAP complex disrupters seem to effectively modulate compartmentalized cardiac cAMP signalling [192,193]. Direct activation of cAMP-generating AC subtypes can be achieved by the cell membrane-permeable diterpene forskolin from the Indian plant *Coleus forskolhlii* [6]. Novel N^6^-derivatives of cAMP, such as 6-Bnz-cAMP, directly and selectively activate PKA [194,195], whereas inhibition of PKA is achieved by H89 and the more selective inhibitors Rp-8-CPT-cAMPS, Rp-cAMPS and Rp-8-Bromo-cAMPS [195,196,197]. Direct activation of Epac (both Epac1 and Epac2) can be achieved by 8-pCPT-2’-*O*-Me-cAMP [194,195,198,199] and the PDE-resistant and cell membrane-permeable Sp-8-pCPT-2’-*O*-Me-cAMP, which exhibits an even increased specificity towards Epac [195,200]. Although pharmacological inhibitors of Epac proteins are not available, *in vitro* down-regulation of Epac expression by silencing RNA provided the first insight into Epac-specific functions [95,107,170,173] (Figure 1). The recently developed Epac1 and Epac2 knock-out mice should be supportive to specifically assign cellular functions to Epac1 and Epac2 [201,202]. These tools allowed studies on the novel aspects of the spatio-temporal nature of (compartmentalized) cAMP signalling in COPD. 

## 3. Cellular Diversity in cAMP Responses: Compartmentalization?

COPD is a chronic inflammatory lung disease mainly caused by cigarette smoking and environmental pollution, and is expected to be the fifth cause of death worldwide by 2020, based on World Health Organization estimates [203]. COPD is characterized by a very slow progressive onset and by respiratory symptoms such as wheezing, cough, chest tightness and dyspnea [26,34,204,205,206]. COPD mainly afflicts middle-aged and elderly people, who usually bear a history of heavy smoking [207,208]. Long-term exposure to smoke (especially cigarette smoke) represents the main risk factor to develop COPD, although less than 25% of smokers develops COPD and at least 15% of COPD-related mortality occurs in never-smokers, suggesting that other factors may be important as well [207,208,209].Cigarette smoke consist of several distinct components such as tar, nicotine, carbon monoxide, ammonia carbonyls, volatiles, semi-volatiles, phenols, aromatic amines and N-nitrosamines [210], and in particular the high level of reactive organic radicals (particle size < 0.5 μm) seems to profoundly disturb functional responses of airway-related cells in the small airways [24,26,211,212]. Cigarette smoke-induced damage of the airway epithelium initiates a chronic cycle of injury and repair that involves the innate immune response, and the recruitment of macrophages as well of neutrophils [213,214]. Such processes contribute to an increase in the neutrophil attractant interleukin-8 and mucus hypersecretion [27,28,30,34,215,216,217] both of which are known to be associated with a higher risk of bacterial or viral infections [28]. Up to 25% of all exacerbations in COPD patients contain the bacteria strains *Haemophilius influenzae* and *Moraxella catarrhalis*, and especially the acquisition of new bacterial strains seems to be important for the onset of exacerbations [34,217]. Mucus hypersecretion and inflammatory mediators not only promote bacterial and/or viral infections, but also enhance inflammatory responses and thereby decrease mucociliarly clearance [34,217]. Thus, cigarette smoke-induced inflammation and mucus hypersecretion most likely promote the development and progression of typical COPD features. 

The accelerated, not fully reversible decline in lung function in COPD is characterized by infiltration and activation of inflammatory cells, particularly macrophages, lymphocytes and neutrophils, which promote the release of proteases and inflammatory cytokines, including interleukin-8 and tumor necrosis factor [24,25,26,204]. Small airways and lung parenchyma are predominantly affected upon inflammation in COPD patients and contribute to the airway obstruction and progressive loss of lung function [34,218]. In addition to inflammatory cells, structural cells, including airway smooth muscle and epithelial cells, underpin a variety of key responses in the airways, such as smooth muscle contraction, airway remodelling, inflammatory cytokine release and mucus hypersecretion, features known to underpin airway obstruction in COPD [24,26,34]. Although the occurrence of airway hyperresponsiveness in COPD is debated, a considerable amount of COPD patients have been shown to exhibit higher responsiveness to contractile stimuli and the severity of airway hyperresponsiveness appears to be a good predictor of the rapid decline in lung function in patients with COPD [219,220,221]. Airway smooth muscle mass increases significantly in the small airways in COPD [222,223,224,225], and this increase is believed to be a main contributor to airway hyperresponsiveness [226].

Airway remodelling typically appears later in adult life [227,228], and predominantly affects small airways and lung parenchyma [227,228]. Airway remodelling in COPD is inextricably linked to the inflammatory cell infiltration into lung tissue and encompasses emphysema, increased mucous, squamous cell metaplasia and increased airway smooth muscle mass, enlargement of the bronchial mucus glands, increased mucus content in the airway lumen, airway fibrosis, and increased epithelial cell proliferation [24]. Vascular remodelling due to inflammatory infiltration of the vessels, is also a characteristic feature of COPD and may generate pulmonary hypertension [229]. An imbalance between proteases (including MMPs) and endogenous antiproteases is likely to be involved in the development of emphysema [230,231,232]. Fibrosis around the small airways is also believed to play a major role in the irreversible airway narrowing in COPD [233]. Airway fibrosis is considered to be the result of an abnormal wound repair mechanism, involving the recruitment and activation of (myo)fibroblasts, which may be derived from resident mesenchymal cells, circulating fibrocytes and epithelial-to-mesenchymal transition, a process in which epithelial cells transdifferentiate into fibroblasts [234,235,236]. Activated fibroblasts produce huge amounts of extracellular matrix proteins like collagens, proteoglycans and glycoproteins like fibronectin and laminins, thus contributing to fibrotic responses in COPD [204,235,237]. In surgically resected lung tissues, increased accumulation of inflammatory exudates with mucus in the small airways was noted to correlate with the severity of disease [23]. In COPD, structural changes include increased and altered extracellular matrix deposition and increased airway smooth mass, increased microvasculature, thickening of the reticular basement membrane, goblet cell metaplasia and epithelial changes [204,238,239,240,241]. Epithelial remodelling driven by cigarette smoke induces epithelial hyperpermeability and the disruption of the epithelial barrier function [27,29], processes known to correlate with goblet cell metaplasia and mucus gland hypertrophy [32,33]. In COPD patients with more severe disease, mucus hypersecretion account for morbidity and mortality [27,34].

Currently, no preventive or curative pharmacological treatment exists for COPD. Airflow obstruction in COPD is predominantly treated with anticholinergics and β_2_-agonists [242,243,244], the latter known to induce bronchodilation by elevating cAMP. β_2_-Agonists effectively reduce airflow obstruction, but have only a poor effect on airway inflammation [204]. Although airway inflammation in asthma can be well controlled by treatment with inhaled glucocorticosteroids in most patients, COPD patients are often characterized by a relative glucocorticosteroid insensitivity [245]. However, β_2_-agonists can augment the anti-inflammatory effect of glucocorticosteroids [246]. Although β_2_-agonists have been shown to inhibit cytokine release *in vitro* [247,248], evidence for their anti-inflammatory properties *in vivo* is still lacking [73]. This discrepancy might be explained by the development of β_2_-adrenoceptor desensitization, particularly in inflammatory cells [249]. Hence, in inflammatory cells inhibitors of phosphodiesterase (PDE) maintain the beneficial effects of β_2_-agonists without the risk of receptor desensitization due to their capacity to elevate cAMP by preventing its breakdown, and control inflammation in the airways and thereby possibly the frequency of exacerbations [125]. Selective inhibitors of PDE4, such as rolipram and roflumilast, have undergone clinical trials to determine their usefulness/efficacy in the treatment of COPD [124]. In contrast to β_2_-agonists, these PDE inhibitors only marginally reduce airflow obstruction [34,124,250,251,252,253]. Notably, though both β_2_-agonists and phosphodiesterase (PDE) inhibitors elevate cAMP, they modulate distinct cellular functions. Compartmentalization of cAMP and its effectors could explain these distinct cellular responses to cAMP by different cAMP elevating drugs. 

## 4. Regulation of Epithelial Barrier Function: Tight Junctions *versus* Adherens Junctions

The following sections will discuss the maintenance of the epithelial barrier in health and disease to maintain proper lung functioning and to alleviate COPD symptoms. Prior to that, we will summarize central structural features of tight and adherens junctions that underpin the maintenance of a proper barrier. Herein, we will mainly focus on the epithelial barrier, and we would like to refer the reader to excellent reviews with focus on the molecular components of the endothelial barrier [254,255,256,257,258,259,260].

**Figure 2:**
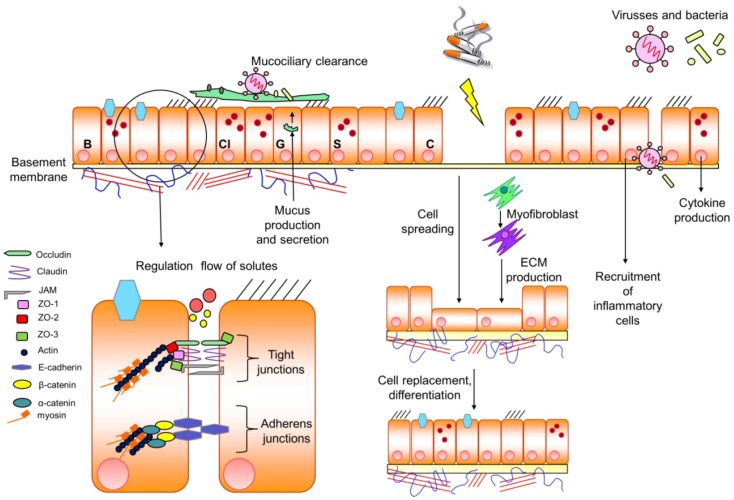
Epithelial barrier functions. Shown are key features of the epithelial barrier under healthy conditions and in the presence of toxic particles and/or infectious agents. Intriguingly, the different cell types composing the epithelial barrier including ciliated (C) cells, basal (B) cells, clara (C) cells and goblet (G) cells exhibit rather diverse functions within the barrier. Ciliated cells are responsible for the mucociliary clearance of infectious agents. Goblet cells produce the mucus needed for the clearance process. Clara cells contain granules filled with antiproteases known to be released by these cells upon their activation. Basement membrane is composed of basal cells known to exhibit next to their structural role a variety of distinct functions within the epithelium (see text for further details). Intercellular cell-cell contact between epithelial cells is achieved by tight junctions and adherens junctions. Adherens junctions ensure a tight adhesion of cells, whereas tight junctions act as a size selective barrier for certain ions and molecules. Exposure of epithelial cells to toxic 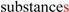 such as cigarette smoke 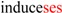 a (persistent) damage of the epithelial barrier, a process being compensated by cell spreading and production of extracellular matrix (ECM) by myofibroblasts to gain cell replacement and differentiation of distinct cells within the epithelium. In addition, toxic particles such as viruses and/or bacteria within the epithelial barrier will induce the recruitment of inflammatory cells and the production of cytokines to diminish the entrance of the devastating particles. For further details see text.

In healthy subjects, the epithelium forms a continuous lining to the airways and to the environment, and play a unique role as a barrier against external deleterious agents by elaboration of a series of defence mechanims developed to protect the airways from insults [212,240,261,262]. The airway epithelium defence system is comprised of several different functions, including structural features of the epithelium that maintain the epithelial barrier integrity, effective mucociliary clearance upon tight regulation of ciliary beating leading to an effective mucociliary clearance, and coordinated regulation of epithelial secretory properties to release molecules with antibacterial, antioxidant, and antiprotease activities [211,236,240,261]. Exposure to cigarette smoke severely alters airway epithelium morphology and function, and subsequently initiates a chronic cycle of injury and repair [29,213,214]. The different types of epithelial cells encompass rather distinct functions within the epithelial cell layer (Figure 2). 

Essentially, the main cell types within the epithelium are divided between secretory and ciliated cells. Epithelial ciliated cells are engaged to transport the mucus out of the airways and thereby to remove the pathogens and toxic particles trapped in the mucus. The functionally close interplay between ciliated and secretory epithelial cells is illustrated by the fact that mucus being utilized by ciliated cells to maintain their proper transport function is produced and secreted by goblet cells, a secretory cell type of the epithelium, whereas a distinct subset of secretory cells release antimicrobial substances as a defence against unwanted pathogens [212,240,261]. The epithelium also consists of basal cells [263,264,265], a separate layer of cells covering most of the airway basal lamina. Due to their central position within the epithelium, basal cells interact with the columnar epithelium (a single cell layer of epithelium cells lining the respiratory tract), neurons, the basement membrane as well as underlying mesenchymal cells, and represent a key component of the epithelial-mesenchymal trophic unit of larger airways [240,264]. Basal cells execute diverse functions within the epithelial cell layer such as the inflammatory response, transepithelial water movement, oxidant defence of the tissue, the formation of the lateral intercellular space and progenitor cell functions for epithelium-associated cells, in particular during the development of the epithelium [263,264,265].

The epithelium is composed of continuous intercellular barriers such as tight junctions known to account for a size-selective barrier of molecules into the epithelium [266]. Tight junctions are located between the apical and lateral cell surface and thereby maintain cell polarity. Tight junctions are characterized by a unique expression profile of proteins such as claudins, occludins, zona occludens and junction adhesion molecules (JAMs) [266,267,268] (Figure 2). Currently, three members of the JAM family of transmembrane proteins have been identified: JAM-A, JAM-B and JAM-C. In particular, JAM-A is highly expressed in the tight junctions of the epithelial cells. In addition, JAM-A controls neutrophil transmigration, primarily across endothelial cells [267,269,270]. As neutrophil numbers seem to be indicative for COPD severity and exacerbation frequency [34,271], it is tempting to speculate that dysfunctions on the level of JAM-A might be also of importance for typical COPD disease features. Interestingly, the coxsackievirus and adenovirus receptor (CAR) represents another member of the JAM family [272,273], and has been reported to regulate the barrier function of tight junctions in epithelial cells [267,274]. 

Zona occludens (ZO) proteins represent other components of the tight junctions [262,275], that regulate junction formation and the interaction with the actin cytoskeleton [276,277,278,279]. Of the three members of the ZO protein family, ZO-1, ZO-2 and ZO-3, ZO-1 exhibits a rather abundant expression profile and binds to myosin, F-actin, ZO-2 and ZO-3 [262,275,280,281] (Figure 2). Studies in Madin-Darby canine kidney cells demonstrated that downregulation of ZO-1 using stable expression of a ZO-1 short hairpin silencing RNA profoundly altered junctional morphology and the organization of the actin cytoskeleton. Decrease of ZO-1 largely reduced the amount of cell-cell contacts and resulted in an intracellular accumulation of actin [282]. Importantly, transepithelial electrical resistance measurements demonstrated that the reduction of ZO-1 expression was paralleled by a reduction of the epithelial barrier as shown by a size selective increase in the movement of molecules < 4 Å through the disrupted barrier [282]. Taken together, ZO-1 controls paracellular permeability by coupling to components of the junctional actin cytoskeleton. Next to the tight junctions, the epithelium consists of an additional intercellular barrier, namely the adherens junctions predominantly expressed at the more basal side of the epithelial cells. Adherens junctions are characterized by the expression of cadherin family members such as E-cadherin and catenin [141,262,275,283]. E-cadherin interacts with α-catenin and/or β-catenin to form adherens junctions [262,275], a process strengthened by connection of adherens junctions to actin filaments present at intracellular sites of the cell-cell contacts [141]. Actin present in epithelial cells will form a circumferential belt which is bound to the adherens junction. Myosin, as part of this belt and bounded to actin, can control the shape of the cell via this belt [240,264,284] (Figure 2). Based on this, myosin fulfils an important function in the molecular architecture of both tight junctions and adherens junctions. As the predominantly expressed protein in the muscle, myosin binds to actin and thereby enables actomyosin-mediated muscle contraction, a process being under control of Rho-Rho-kinase [262,268,285,286]. The phosphorylation of the Rho-Rho-kinase target myosin light chain is decreased due to a reduction in the RhoA/Rac1 ratio via cAMP-driven Epac activation. Dephosphorylation of the myosin light chain relaxes the smooth muscle [87,88]. Myosin light chain, not only found in smooth muscle cells, but also in epithelial cells can be phosphorylated by Rho-Rho-kinase, increasing the epithelial barrier. 

From the multiple myosin isoforms, myosin IIA and IIB are primarily expressed at cell-cell contacts [287]. Importantly, is has been reported recently that myosin IIA and myosin IIB engage rather distinct signalling cascades to regulate cadherin junctions in MCF7 breast epithelial cells [142]. Junctional localization of myosin IIA requires next to E-cadherin adhesion, Rho-Rho-kinase and myosin light chain-kinase activation, and thereby subsequently increase the contractile force of the circumferential belt and tight junction integrity. Myosin IIB, via Rap1A, supports myosin IIA-Rho-Rho-kinase signalling to E-cadherin and to myosin light chain kinase, and thereby also subsequently induce the stabilisation of the apical ring structure and enhancement of the junctional integrity [142]. Thus, both myosin IIA and myosin IIB might modulate the epithelial barrier function upon enhancement of the (tight) junction integrity through signalling via a rather distinct subset of small GTPases, namely RhoA and Rap1A. Several recent reports indicate that Rap1 regulates cell-cell junction formation through signalling to E-cadherin-catenin and integrin-extracellular matrix complexes [22,151,288,289]. Thus, it is tempting to assume that Epac, by activation of Rap1, importantly regulates the barrier function. As it has been also shown that E-cadherin internalization induces GTP-loading, thus, activation of Rap1 [140], these findings might indicate that the activity state of Rap1 is not only controlled by guanine nucleotide exchange factors but also by structural components of the cell-cell barrier.

The next layer of the epithelial barrier, the basement membrane belongs to the basement membrane zone and is a central component of the epithelial mesenchymal trophic unit, the latter known to consist of opposing layers of epithelial and mesenchymal cells separated by the basement membrane [240,263,264,265]. The basement membrane executes several distinct functions such as epithelium-extracellular matrix attachment, barrier functioning, cell-cell/cell-matrix communication and binding of growth factor, including the epidermal growth factor and the transforming growth factor-β (TGF-β) [240,263,264,265] (Figure 2). Although thickening of the epithelial reticular basement membrane is a typical feature of asthma [228], structural alterations on the level of the basement membrane and its potential impact on typical COPD features is still a matter of debate. The extracellular matrix underneath the basement membrane consists of several distinct components such as collagens, proteoglycans and glycoproteins like fibronectin and laminins [212,261,262,263,264]. In COPD patients a decrease in proteoglycans, such as decorin and biglycan, is observed. Collagen and fibronectin are increased in patients with emphysema leading to airway wall fibrosis [204,238,240,264,284,290].

Activated fibroblasts produce huge amounts of the extracellular matrix components, but also regulate the production of MMPs and endogenous antiproteinases [291,292,293]. Elevation of TGF-β promotes cell proliferation and extracellular matrix deposition by fibroblasts, whereas MMP-2 and MMP-9 (gelatinase / collagenase) and MMP-12 (elastase), which are increased in sputum of COPD patients [204,294] promote pro-fibrotic responses as well as destruction of the alveolar parenchyma. Thus, an imbalance between matrix metalloproteinase and their appropriate antiproteinase may contribute to fibrosis and emphysema in COPD [204,294,295,296,297]. 

**Figure 3:**
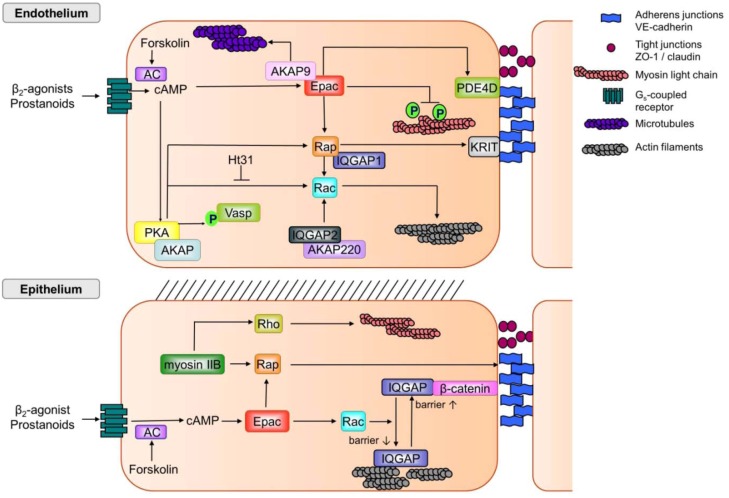
cAMP signalling in endothelial cells *vs.* epithelial cells. β_2_-agonists and prostanoids which activate their appropriate G-protein coupled receptor, and forskolin which activates adenylcyclase (AC) will increase cAMP production in both endothelial and epithelial cells resulting in cell type specific responses in both cells. In endothelial cells, cAMP increase will cause activation of both PKA and Epac. Activation of Epac will enhance microtubule growth which is AKAP9-dependent [148]. Epac activation will result in binding to PDE4D (phosphodiesterase 4D) which binds to the E-cadherin complex causing improvement of the barrier [147]. Next to this, Epac activation will reduce the phosphorylation of the myosin light chain, causing relaxation and improvement of the barrier [87,88]. The other effector of cAMP, PKA, anchored to AKAP (A-kinase anchoring protein) will activate both Rap and Rac. Active Rap, stabilized by IQGAP1, can activate KRIT which stabilizes cell-cell contacts. IQGAP2, bound to AKAP220, mediates calcium-dependent Rac activation which can alter actin dynamics [143,144,145,302,303]. In epithelial cells, cAMP elevation will activate Rap and Rac via Epac. Rac in its active state will reduce the binding of IQGAP1 to β-catenin, resulting in a decrease of the epithelial barrier [141]. Rap is also activated by myosin IIB and will thereby enhance the barrier via E-cadherin. Myosin IIB will also activate Rho-Rho-kinase which via myosin light chain phosphorylation will increase the barrier properties [142].

Cigarette smoke belongs to the major risk factors of COPD [32,298], and is known to induce a vicious cycle of injury and repair in the airway epithelium upon adaptation on the level of tight junction and adherens junction morphology and function [31,212,213,214], a process which may eventually end up in transcriptional reprogramming of the airway epithelium [29]. Unfortunately, cigarette smoke-induced repair mechanism may also worsen airway obstruction of COPD patients through mucus hypersecretion by goblet cell hyperplasia, down-regulation of epithelial ciliated cells and hypertrophy of the submucosal gland [24,204,212,213,261]. Importantly, a recent study of Crystal and colleagues demonstrated that cigarette smoke exposure of airway epithelium induced a profound down-regulation of the majority of the typical tight junction and adherens junctions components including claudins, ZO proteins, E-cadherin and catenin [29]. In contrast, cigarette smoke exposure induced a profound up-regulation of molecular pathways known to be critical for epithelial differentiation including the phosphatase and tensin homolog (PTEN), phosphoinositide 3-kinase-dependent PKB/Akt, the cAMP effector PKA and Rac [29]. As several recent studies indicate that a proper signal balance between RhoA *versus* Rac1 seems to be of utmost importance for the regulation of both the endothelial and epithelial barrier [2,112,153,299,300,301], a subtle interplay between the cAMP-effector Rap1 and Rac1 might be envisioned as a key event to preserve the structure and function of tight and adherens junctions (Figure 3). Particularly, the impact of Rac1 on the endothelial barrier seems to be rather puzzling as both stabilization and destabilization of the endothelial barrier integrity have been observed [147,152,153,154,155,156,157] (Figure 2 and Figure 3). Intriguingly, these opposing findings might be partly explained by the distinct barrier protection properties of Rac1 in micro-vascular *versus* macro-vascular endothelial cells. Future studies should intend to unravel the precise underlying molecular signalling pathways and thereby to develop novel therapeutical interventions to restore and to maintain proper epithelial barrier in patients with COPD.

In epithelial cells, cAMP elevation will activate Rap and Rac via Epac. Rac in its active state will reduce the binding of IQGAP1 to β-catenin, resulting in a decrease of the epithelial barrier [141]. Rap is also activated by myosin IIB and will thereby enhance the barrier via E-cadherin. Myosin IIB will also activate Rho-Rho-kinase which via myosin light chain phosphorylation will increase the barrier properties [142].

## 5. Novel Aspects of Barrier Functioning

Insights into the molecular mechanisms being implicated in the maintenance or even restoration of the epithelial barrier function are still rather limited, therefore we will outline the molecular mechanisms that maintain the endothelial barrier function in the vasculature, we will translate these findings onto the regulation of the epithelial barrier function in COPD. 

It is generally accepted that several endothelial barrier disrupting agents, such as tumor necrosis factor-, thrombin and the bacterial endotoxin lipopolysaccharide, profoundly alter the molecular architecture as well as the dynamics of the actin-microtubule network known to comprise the barrier tight and adherens junctions [258,259,260,261,262]. Importantly, cAMP elevating agents such as β_2_-agonists, prostanoids (prostacyclin, prostaglandin, PGE_2_) and the direct AC activator forskolin effectively reduce the leakage of the endothelial barrier in whole animals, isolated lungs and in clinical studies under both resting conditions and exposure to inflammatory mediators [2,304,305,306]. Several conclusive studies by Waschke and colleagues [153,156,157,307] demonstrated that at least part of the cAMP-dependent enhancement of the human dermal microvascular endothelial barrier (measured by transelectrical resistance (TER)) is mediated via the activation of Rac1 by both vasodilator-stimulated phosphoprotein (VASP) and AKAP-anchored PKA (Figure 3). Activation of Rac1 by AKAP-anchored PKA was sensitive to the PKA-AKAP-binding blocking peptides st-Ht31 [158]. As shown in human lung microvascular endothelial cells [308], PKA-dependent phosphorylation of VASP might also contribute to the barrier protection. 

Although the members of the AKAP superfamily involved in the barrier function of human dermal microvascular endothelial cells still have to be identified, these results indicate that compartmentalized cAMP signalling by AKAPs contribute to the endothelial barrier function. The magnitude of the cAMP-dependent endothelial barrier maintenance, a process being paralleled by the subcellular localization of endothelial adherens junction marker VE-cadherin [153,156,157], profoundly differs between the β_2_-agonist epinephrine, the direct AC activator forskolin and the PDE4 inhibitor rolipram, the latter being non-effective on its own and acquiring biological effectiveness only in the presence of forskolin. These findings indicate that forskolin and rolipram are functionally different and/or require a distinct assembly of cAMP signalling pools. Indeed, studies from Maurice and colleagues demonstrated that PDE4D-bearing VE-cadherin-based multiprotein complexes control the vascular permeability in HUVECs [147,155]. Next to PDE4D and VE-cadherin, cAMP-dependent Epac1 was shown to be vital for the regulation of the endothelial barrier in HUVECs [147]. Indeed, subsequent studies by Waschke and colleagues indicated that the direct Epac activator 8-pCPT-2'-*O*-Me-cAMP mimicked the effect of epinephrine and forskolin on the barrier function and subcellular distribution of VE-cadherin in human microvascular endothelial cells [153,157]. As shown before for VASP and AKAP-anchored PKA, direct activation of Epac induced also the GTP-loading of Rac1 [153,157]. In addition, Hordijk and colleagues reported that Rac1 induced the production of reactive oxygen species and subsequently induced subcellular redistribution of VE-cadherin-catenin complexes in HUVECs [309]. Such mechanisms might worsen the endothelial barrier function under inflammatory disease conditions (Figure 3).

Interestingly, protection of the endothelial barrier upon activation of Rac1 was restricted to microvascular endothelial cells [157]. It is tempting to speculate that a distinct subset of cAMP-sensing AKAP-bearing multiprotein complexes might be expressed in micro-vascular *versus* macro-vascular endothelial cells. In support, recent studies point to the existence of a rather heterogeneous composition of such multiprotein complexes in cardiomyocytes [79], neurons [19], human lung fibroblasts [82,122] and airway smooth muscle [117]. Such diversity may also -at least in part- explain the opposing effects on the endothelial barrier – both protection and disruption - observed upon β_2_-adrenegic receptor stimulation [153,156]. 

Birukova and colleagues characterized the molecular mechanisms leading to Rac1 activation by Epac1 in human pulmonary artery endothelial cells *in vitro* and in ventilator-induced lung injury *in vivo* [146,310,311,312,313]. Elevation of cellular cAMP content, *e.g.*, by prostaglandin E_2_ and prostacyclin I_2_, induced GTP-loading of Rac1 via Epac1-dependent Rap1 activation and the engagement of the Rac-specific GEFs Tiam1 and Vav2, processes being supported by PKA and the inhibition of p115 Rho-GEF-dependent activation of RhoA. Altogether, these mechanisms contribute to the barrier protection observed in human pulmonary artery endothelial cells *in vitro* and to the attenuation of ventilator-induced lung injury *in vivo* [146,310,311,312,313]. As reported for the molecular mechanisms leading to the relaxation of smooth muscle [87,88], Epac1 most likely protects the endothelial barrier by decreasing the phosphorylation of the Rho-Rho-kinase target myosin light chain by skewing the balance of RhoA/Rac1 activation towards Rac1. These recent findings confirmed initial studies by the research groups of Mayadas [148,314] and Mochizuki [22,314], reporting on the first molecular link of Epac and the actin-microtubule, and its impact on the regulation of the barrier function in HUVECs and human pulmonary aortic endothelial cells. Recently, Ginsberg and colleagues demonstrated that Krit1 (Krev1 interaction trapped gene) is required for the stabilization of β-catenin-bearing cell-cell contacts by the Epac effector Rap1, and that the Epac-Rap1 effector Krit1 is required for the maintenance of the endothelial barrier [315,316]. Loss of Krit1, known to account for the loss of endothelial junctions in cerebral cavernous malformations [316], induces destabilization of the endothelial barrier by increasing the phosphorylation of the Rho-Rho-kinase target myosin light chain. Although the involvement of Epac has not been studied by Ginsberg and colleagues [316], these findings might indicate that Epac-bearing multiprotein complexes are of utmost importance to maintain the endothelial barrier properties (Figure 3).

Intriguingly, Mayadas and colleagues reported recently that AKAP9 induced Epac1-dependent-microtubule growth resulting in stabilization of the barrier function in HUVECs and human dermal microvascular endothelial cells [149]. As AKAP9 has been reported to bind to PDE4 [123], PDE4D-dependent Epac1 binding to VE-cadherin-based signalling complexes might contribute to the maintenance of the micro-vascular endothelial permeability [147]. This process which might be supported by an Epac1 dependent enhancement of cell-cell adhesion and integrin-extracellular matrix interactions [22,151]. As Waschke and colleagues reported recently on the activation of Rac1 by AKAP-anchored PKA [158], it is tempting to speculate that the AKAP9 mediates the Epac-dependent Rac1 activation in human micro-vascular endothelial cells. In addition, it has been reported that a GTPase-deficient mutant of IQGAP1 induced GTP-loading of Rac1 and inhibited IQGAP1 sequestration of β-catenin, and thereby subsequently stabilized E-cadherin-dependent barrier function of MCF7 breast epithelial cells which show epithelium characteristics [143,144,145]. As IQGAP1 binds also to the Epac-effector Rap1 [143], Epac-dependent compartmentalized cAMP signalling in human micro-vascular endothelial cells might require next to AKAP superfamily members IQGAPs. In line with this assumption, Scott and colleagues demonstrated recently that a calcium-dependent AKAP220-IQGAP2 complex mediated Rac activation and thereby cellular actin remodelling [143,302,303]. The existence of such AKAP-IQGAP complexes in the endothelium and their contribution to the regulation of the endothelial barrier still has to be studied. 

Although it is generally accepted that cigarette smoke alters the epithelial functions in COPD remodelling [27,28,29,30,31,32,33], the molecular mechanisms contributing to the regulation of the epithelial barrier are by far less characterized compared to several recent studies with focus on the endothelial barrier. Little information is known on the epithelial barrier in COPD, but studies performed in other types of epithelial cells may indicate a potential role of cAMP signalling pathway in the restoration of the barrier in COPD patients. 

It has been reported for podocytes (renal glomerular visceral epithelial cells) that the AC activator forskolin induced a redistribution of ZO-1, E-cadherin, and β-catenin to cell-cell contacts [317]. On the other hand, reduction of cellular cAMP levels upon PDE inhibition by pentoxifylline attenuated tight junctions of immunostimulated Caco-2 human intestinal epithelial cells [318]. 

Importantly, Menke and colleagues reported recently that transformation of the human pancreatic carcinoma epithelial-like cell line PANC-1 with constitutively active Rac1(V12) profoundly altered the subcellular distribution of E-cadherin- β-catenin complexes and thereby epithelial cell-cell contacts in an IQGAP1-dependent manner, whereas cell transformation with dominant negative Rac1 (N17) had the opposite effect [141]. Together with the finding that in MCF7 breast epithelial cells myosin IIB signals via Rap1A to E-cadherin and to the Rho-Rho-kinase effector myosin light chain and subsequently enhance junctional integrity [142], a diligent balance of the GDP/GTP-loading of the small GTPase superfamily members Rac1 and Rap1 -most likely driven by compartmentalized cAMP signalling by a distinct subset of AKAPs and IQGAPs- seem to be of key importance to maintain a proper barrier function in epithelial cells. Of interest to note is that IQGAP1 and IQGAP2 seem to be differentially involved in the regulation of the cellular barrier [143,144,145] and the actin-microtubule network dynamics [302,303]. Indeed, recent research indicate also that IQGAP1 and IQGAP2 fulfill distinct functions in tumorigenesis, whereas IQGAP1 acts as an oncogene IQGAP2 seems to act as a tumor suppressor (Figure 3) [319]. Preliminary results of our group show that cigarette smoke-mediated disruption of human bronchial epithelial (HBE) barrier correlates with a down-regulation of AKAP9 [130]. As AC9 is the main isoform in bronchial epithelial cells [123,136], it is tempting to speculate that the previous described interaction between AC9 and AKAP9/Yotiao [127] is involved in this process. Next to AKAP9, AKAP5 and AKAP12 are also expressed in human bronchial epithelial cells, but their expression is not sensitive to cigarette smoke exposure. Importantly, the expression of AKAP9 mRNA was also down-regulated in primary epithelial cells of current smokers compared to non/ex-smokers as well as in lung biopsies from COPD patients [320]. 

Taken together, compartmentalized cAMP signalling maintained by a distinct subset of cAMP-responsive multi-protein complexes seen also to account for the proper functioning of the epithelial barrier, the latter known to be derailed in patients with COPD. Future research should aim to target cell-type specific cAMP-sensing complexes to augment our current therapeutically treatment regimes for chronic inflammatory disorders such as COPD.

## 6. Conclusions

G-protein-coupled receptors, adenylyl cyclases and PDEs regulate, in a spatio-temporal manner, the cellular cAMP concentration and subsequent cAMP signalling. Compartimentalization of cAMP signalling through a distinct subset of multi-domain proteins of the AKAP family supports fine-tuning of the net-outcome of cAMP-regulated cellular responses. Novel insights into cAMP compartmentalization upon manipulation of these multi-protein complexes may lead to new therapies in diseases like heart failure, cancer and COPD, known to be characterized by cAMP dysfunction. 

In COPD, dysfunction of the epithelial barrier results in progression of disease symptoms. Since cAMP exhibits protection of the barrier function in endothelial cells, targeting the cAMP pathway may also restore the damaged epithelial barrier in COPD. Given the importance of compartmentalized cAMP signalling in regulating cellular barrier functions, alterations in maintenance of the protein-protein communication may lead to the observed barrier dysfunction in COPD. Upon activation of Rap and inhibition of Rho, the cAMP effectors Epac and PKA increase cellular barrier function.

Future studies with focus on cAMP compartmentalization will be required to further unravel the underlying molecular mechanisms. Further understanding of this compartmentalized cAMP signalling will be of benefit for improvement of the current therapeutic arsenal for the treatment of COPD.
